# Divergent Phenotypes in Mutant TDP-43 Transgenic Mice Highlight Potential Confounds in TDP-43 Transgenic Modeling

**DOI:** 10.1371/journal.pone.0086513

**Published:** 2014-01-22

**Authors:** Simon D’Alton, Marcelle Altshuler, Ashley Cannon, Dennis W. Dickson, Leonard Petrucelli, Jada Lewis

**Affiliations:** 1 Department of Neuroscience and Center for Translational Research in Neurodegenerative Disease, University of Florida, Gainesville, Florida, United States of America; 2 Department of Neuroscience, Mayo Clinic, Jacksonville, Florida, United States of America; International Centre for Genetic Engineering and Biotechnology, Italy

## Abstract

The majority of cases of frontotemporal lobar degeneration and amyotrophic lateral sclerosis are pathologically defined by the cleavage, cytoplasmic redistribution and aggregation of TAR DNA binding protein of 43 kDa (TDP-43). To examine the contribution of these potentially toxic mechanisms *in vivo,* we generated transgenic mice expressing human TDP-43 containing the familial amyotrophic lateral sclerosis-linked M337V mutation and identified two lines that developed neurological phenotypes of differing severity and progression. The first developed a rapid cortical neurodegenerative phenotype in the early postnatal period, characterized by fragmentation of TDP-43 and loss of endogenous murine Tdp-43, but entirely lacking aggregates of ubiquitin or TDP-43. A second, low expressing line was aged to 25 months without a severe neurodegenerative phenotype, despite a 30% loss of mouse Tdp-43 and accumulation of lower molecular weight TDP-43 species. Furthermore, TDP-43 fragments generated during neurodegeneration were not C-terminal, but rather were derived from a central portion of human TDP-43. Thus we find that aggregation is not required for cell loss, loss of murine Tdp-43 is not necessarily sufficient in order to develop a severe neurodegenerative phenotype and lower molecular weight TDP-43 positive species in mouse models should not be inherently assumed to be representative of human disease. Our findings are significant for the interpretation of other transgenic studies of TDP-43 proteinopathy.

## Introduction

TAR DNA binding protein of 43 kilodaltons (TDP-43) is the major pathological substrate in frontotemporal lobar degeneration with TDP-43 pathology (FTLD-TDP) and most cases of amyotrophic lateral sclerosis (ALS) [1]. TDP-43 is a member of the hnRNP family, containing two RNA Recognition Motifs (RRMs) that bind RNA and a C-terminal, glycine rich domain that mediates interactions with functional binding partners to coordinate the metabolism of a wide range of RNA substrates [2], [3]. In disease, normal nuclear localization of TDP-43 is lost, and ubiquitinated and hyperphosphorylated inclusions are deposited in the form of neuronal intranuclear inclusions or in the cytoplasm as juxtanuclear aggregates or dystrophic neurites [4]. TDP-43 also undergoes processing to produce C-terminal fragments that range in size from 15–35 kDa [1], [5], [6]. Evidence for definitive pathological involvement in disease is additionally derived from causal familial mutations in the gene encoding TDP-43, *TARDBP*, in ALS, which are principally though not exclusively found in the glycine rich C-terminus [7].

It is unclear if one or several of these pathological TDP-43 alterations is a dominant driving mechanism in disease, and there is evidence to support the toxicity of each in FTLD-TDP. Pathological C-terminal fragments of the protein are more aggregate-prone, undergoing phosphorylation, ubiquitination and ultimately demonstrating cytotoxicity in cells [8], [9], [10]. Expression of TDP-43 containing disease linked mutations found in ALS appears to increase the production of C-terminal fragments, aggregation and toxicity [9], [11], [12]. Loss of TDP-43 also appears to be deleterious to cell health; knockdown *in vitro* confers toxicity and knockout results in lethality *in vivo*, both *in utero* in constitutive *Tardbp^−/−^* mice and in conditional knockout animals in which deletion is postponed until adulthood [13], [14], [15], [16]. Loss of TDP-43 specifically in motor neurons results in cell death and an ALS-like phenotype in mice [17] and reduced TDP-43 expression in zebrafish and drosophila results in motor deficits [18], [19].

To determine if one mechanism is more dominant than others *in vivo*, a host of constitutive and inducible transgenic rodents expressing human or mouse (wild type and mutant) TDP-43 under the control of various promoters have been created, which recapitulate some of the hallmark pathology of FTLD-TDP/ALS including inclusions of ubiquitinated, phosphorylated TDP-43 and TDP-43 immunoreactive lower weight molecular species [20], [21], [22], [23], [24]. Here we describe the pathological and biochemical characterization of two novel lines of mice conditionally expressing human mutant M337V TDP-43, providing further insight into the nature of neurodegenerative phenotypes in TDP-43 expressing animal models.

## Methods

### Ethics Statement for Animal Care

All procedures were conducted according to the National Institutes of Health guide for animal care and approved by the Institutional and Animal Care and Use Committee at University of Florida or Mayo Clinic.

### Generation of Transgenic iTDP-43 Animals

iTDP-43 mice were generated similarly to a previously described protocol [25]. Full length, untagged, M337V human TDP-43 cDNA was created using the Quickchange II site directed mutagenesis kit (Stratagene) using a TDP-43-myc plasmid as a template [26], and was inserted into the inducible expression vector pUHD 10–3 containing five tetracycline operator sequences. The construct was confirmed by restriction enzyme digest and direct sequencing. The transgenic fragment was obtained by *BsrBI* digestion, gel purified followed by β–agarase digestion (NEB), filtration and concentration. The modified TDP-43 transgene was injected into the pronuclei of donor FVB/NCr embryos (Charles River). 14 founders were positive for the TDP-43 responder transgene. These were then bred with 129S6 mice (Taconic) with the tetracycline transactivator (tTA) transgene downstream of calcium calmodulin kinase II alpha (CaMKIIα) promoter elements [27] to produce the iTDP-43 transgenic mice with forebrain hTDP-43 expression. 8 founders transmitted and expressed the TDP-43 transgene and we subsequently chose the two founder lines with the highest transgenic TDP-43 expression at 2 months of age. All experimental mice used in this study were F1 hybrids produced from a breeding of TDP-43 monogenic mice on a congenic FVB/Ncr and tTA monogenic mice on a 129S6 background.

### Antibodies

Antibodies used in this work are described in table s1. Epitopes recognized by TDP-43 antibodies are detailed in figure s1.

### Immunohistochemistry

After euthanasia via cervical dislocation, brains were harvested and divided along the midline. The right hemisphere was flash-frozen on dry ice, while the left hemisphere was drop fixed in 10% neutral buffered formalin for histological analyses. Brains were embedded in paraffin and cut into 5 µm sagittal sections. For hTDP-43, caspase 3 and ubiquitin antibodies, tissues were immunostained using the DAKO Autostainer (DAKO Auto Machine Corporation, Carpinteria, CA) with DAKO Envision HRP System. For p403/404 detection, sections were deparaffinzed and hydrated through a graded alcohol series prior to antigen retrieval in citrate buffer (10 mM sodium citrate, 0.05% Tween, pH 6.0) for 30 minutes in a steamer with water at a rolling boil. Blocking was performed using DAKO Protein Block for 1 h followed by incubation overnight with primary antibody at 4°C. Peroxidase conjugated secondary antibody was visualized with diaminobenzidine (Vector Labs, Burlingame, CA). Hematoxylin and eosin (H&E) staining was performed by standard procedures.

### Immunofluorescence

Sections were prepared as above, except visualization of primary antibody was performed using Alexa Fluor 488 secondary antibody (Invitrogen). Slides were dipped in Sudan Black to reduce background autofluorescence and vectashield with DAPI (Vector Laboratories) was used to stain nuclei. Images were captured using an Olympus BX60 microscope (Olympus).

### Protein Isolation, Fractionation and Western Blotting

To isolate SDS-soluble fractions, brain or dissected cortex was homogenized in lysis buffer (50 mM Tris, 300 mM NaCl, 1% Triton X-100, 1 mM EDTA with protease inhibitors and phosphatase inhibitors (Sigma)) at 6 ml/g tissue, and aliquots of homogenate stored at −80°C. Lysate was prepared via brief sonication, addition of SDS to 1% and centrifugation at 40K×*g* for 20 minutes at 4°C. For experiments using HEK293T cells, cells were washed once with and then harvested in ice cold PBS. Cells were then pelleted by brief centrifugation at 500×*g* and lysed in lysis buffer as described above. 30–50 µg protein was loaded onto 10% or 15% Tris–glycine polyacrylamide gel (Novex). Following electrophoresis and transfer, nitrocellulose membranes were blocked with 5% milk in TBS-T (Tris-buffered saline with 0.1% Triton X-100), incubated with appropriate primary and HRP-conjugated secondary antibodies and visualized using ECL reagent (Perkin-Elmer). Images were captured on the ProteinSimple FluorChem E (ProteinSimple, Santa Clara, California). In all cases where blotting was to be quantified, lysate prepared fresh from homogenate was used, and following probing with antibody of interest the blot was stripped (70 mM SDS in Tris HCl (pH 6.8) with 0.7% BME for 30 minutes at 55°C) and re-probed with loading antibody. Densitometry was performed using AlphaView (ProteinSimple), and following normalization to loading controls, fold changes calculated and unpaired *t-*test used to assess significance.

To analyze solubility of TDP-43 fragments, cortex from P5 animals was homogenized in 5 ml/g of high salt buffer (10 mM Tris pH 7.5, 5 mM EDTA, 10% sucrose, 1% triton, 0.5 M NaCl, with protease and phosphatase inhibitors), incubated at 4°C for 15 minutes and clarified by centrifugation at 110K×*g.* The supernatant containing the high salt fraction was snap frozen on dry ice and the pellet washed and re-homogenized in 5 ml/g myelin floatation buffer (high salt buffer with 30% sucrose). This homogenization, centrifugation and fraction collection sequence was repeated with sarkosyl buffer (high salt buffer substituting 1% triton for 1% N-lauroyl-sarcosine, incubation 1 hour at room temperature), and finally in 1 ml/g urea buffer (30 mM Tris pH 8.5, 4% CHAPS, 7 M urea, 2 M thiourea, brief sonication). 50 ug of high salt extract and equivalent volumes of all other fractions underwent Western blotting as described above.

### Quantitative Real Time PCR

Total RNA was isolated from dissected hippocampus or cortex using TRIzol reagent (Life Technologies) and Pure Link RNA Mini Kit (Life Technologies). During this step, DNA was removed using on-column DNase digestion (Life Technologies) and the resulting RNA purity and integrity was assessed on spectrophotometer (Nanodrop, Wilmington, USA) and agarose gel electrophoresis. 2 µg of RNA was used to synthesize cDNA using the High Capacity cDNA Reverse Transcription Kit (Applied Biosystems). All samples were run in triplicate on the ABI 7900 HT Real-Time PCR Detection System using SYBR green PCR master mix (Applied Biosystems). Primer efficiencies (E) were calculated using E = 10^(−1/slope)^, where slope was determined by plotting the Cq (quantification cycle) values against the log_10_ input of a cDNA dilution series. A 1/50 dilution of each experimental cDNA sample was run. From this, the relative quantities (RQ) were calculated for each primer pair in each sample and normalization of *Tardbp* performed using the geometric mean of the two reference genes *Gapdh* and *β-actin,* giving the Normalized Relative Quantity (NRQ) [28]. Primer sequences for qPCR were *Tardbp*1 F (5′AAAGGTGTTTGTTGGACGTTGTACAG 3′), *Tardbp*1 R (5′ AAAGCTCTGAATGGTTTGGGAATG 3′), *Tardbp*2 F (5′ GATTGGTTTGTTCAGTGTGGAGTATATTCA 3′), *Tardbp*2 R (5′ ACAGCAGTTCACTTTCACCCACTCA 3′), *Tardbp*3 F (5′ GGTGGTTAGTAGGTTGGTTATTAGGTTAGGTA 3′), *Tardbp*3 R (5′ AAATACTGCTGAATATACTCCACACTGAACA 3′), *Gapdh* F (5′ CATGGCCTTCCGTGTTCCTA 3′), *Gapdh* R (5′ CCTGCTTCACCACCTTCTTGAT 3′), *β-actin* F (5′ GATGACCCAGATCATGTTTGAGACCTT 3′) and *β-actin* R (5′ CCATCACAATGCCTGTGGTACGA 3′). Single PCR products were verified by melt curve analysis. Statistical significance was assessed using unpaired t-test.

### TDP-43 Plasmid Generation

Full-length human TDP-43 complementary cDNA in plasmid pEGFP-C1 [10] was used as PCR template to generate N-terminally myc tagged TDP-43^208–414^ and TDP-43^1–280^. The primers used were TDP-43^208–414^ F (5′-TACGGATCCCACCATGGAACAAAAACTCATCTCAGAAGAGGATCTGCGGGAGTTCTTCTCTCAGTACGG-3′); TDP-43^208–414^ R (5′- GAATCGCGGCCGCCTACATTCCCCAGCCAGAAGACT-3′); TDP-43^1–280 ^F (5′-TACGGATCCCACCATGGAACAAAAACTCATCTCAGAAGAGGATCTGATGTCTGAATATATTCGGGTAACCGAA-3′); TDP-43^1–280 ^R (5′-GAATCGCGGCCGCTCATGGATTACCACCAAATCTTCCAC-3′). Products were cloned into pcDNA3.1 (Life Technologies) using BamH1 and Not1 sites and plasmids were verified by sequence analysis.

### Cell Culture and Transfection

Human Embryonic Kidney 293T cells were maintained in Dulbecco’s Modified Eagle’s Medium (Lonza) supplemented with 10% Fetal Calf Serum (Sigma) and penicillin-streptomycin (Life Technologies). Transfection in 6 well plates was performed for 48 hours using 2.0 ug of plasmid and Lipofectamine 2000 (Life Technologies) following the manufacturer’s guidelines.

## Results

### Early, Rapid Postnatal Cell Loss Induced by hTDP-43^M337V^ Prevents Brain Development without Causing TDP-43 Aggregation

**Figure 1:**
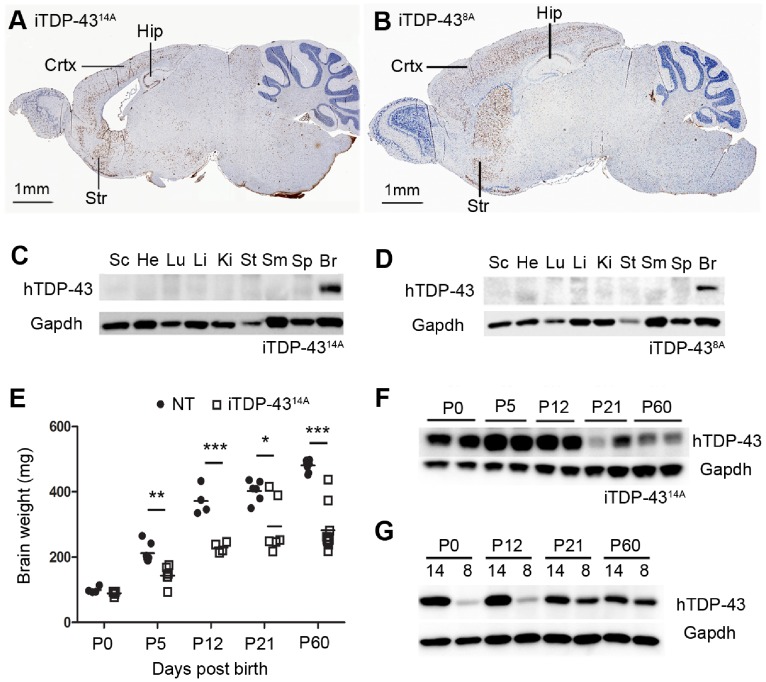
Expression of human TDP-43 in iTDP-43^14A^ and iTDP-43^8A^ mice in the postnatal period. Immunohistochemical detection of hTDP-43 expression in cortex (CTX), hippocampus (HIP) and striatum (STR) in iTDP-43^14A^ (A) and iTDP-43^8A^ (B). Western analysis of organs demonstrated specificity of hTDP-43 expression to the brain in both iTDP-43^14A^ (C) and iTDP-43^8A^ (D) (SC = spinal cord, He = heart, Lu = lung, Li = liver, Ki = kidney, St = stomach, SM = skeletal muscle, Sp = spleen, Br = brain). (E) Brain weight measurement of non-transgenic (NT) and iTDP-43^14A^ mice at postnatal stages until 2 months of age (P60) (*p<0.05, **p<0.01, *** p<0.001, unpaired two tailed *T-test*). (F) Expression of hTDP-43 at indicated postnatal time points for iTDP-43^14A^. (G) Expression of hTDP-43 at indicated postnatal time points for iTDP-43^14A^ (14) compared to iTDP-43^8A^ (8).

Fourteen monogenic, transgenic M337V hTDP-43 founder lines were bred to animals expressing the tetracycline transactivator (tTA) to produce bigenic iTDP-43 mice expressing human mutant TDP-43. We initially screened iTDP-43 mice at two months of age for expression of human TDP-43 and selected the two highest expressing lines (14A and 8A) for further analysis. Transgene expression in bigenic animals from both lines was limited exclusively to the brain, predominantly in the cortex, hippocampus and striatum (figure 1A–D), consistent with what has been previously reported with this conditional system [24], [27]. Phenotypically these animals did not display any premature death or overt signs of neurological dysfunction as we and others have reported in TDP-43 transgenic animals [22], [24], [29]. However, while iTDP-43^8A^ animals developed normal brain structure, there was obvious reduction in the cortical volume of iTDP-43^14A^ brains compared to non-transgenic (NT) littermates (figure 1A).

To fully characterize the progression of cortical degeneration in iTDP-43^14A^, we examined postnatal ages from P0 to P60 to determine the time point of initial phenotypic onset. During this period, brain weights of NT mice experienced a rapid phase of growth between P0 and P12, followed by more modest increases into adulthood (figure 1E). iTDP-43^14A^ mice however demonstrate striking abnormalities in brain weight during postnatal development. There was no difference in gross brain weight of iTDP-43^14A^ mice compared to NT mice at P0; however, iTDP-43^14A^ brain weight was reduced by 33% (NT = 212 mg±11, iTDP-43 = 143 mg±11, *p* = 0.002) by P5 and iTDP-43^14A^ brain weight never reached that of non-transgenic litter mates (figure 1E). This phenotype was not observed in either monogenic tTA or monogenic hTDP-43^14A^ mice, and thus is a result of hTDP-43 expression in bigenic animals (figure s2A). Analysis using a human specific TDP-43 antibody revealed large changes in observed transgene expression over this time period. Peak transgene expression at P5 coincided with age of phenotypic onset before gradual reduction to the levels observed at P60 (figure 1F). Although iTDP-43^14A^ and iTDP-43^8A^ mice expressed similar levels of human M337V TDP-43 in the initial screen at P60, transgene expression in iTDP-43^8A^ mice at earlier time points was far lower than in iTDP-43^14A^ mice, likely explaining the phenotypic difference between the two lines (figure 1G).

**Figure 2:**
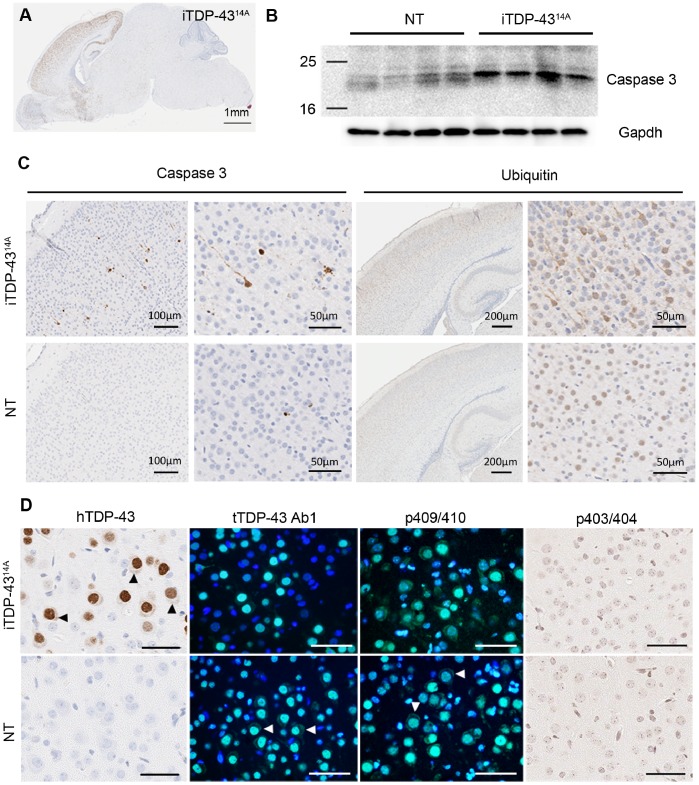
Early degenerative phenotype in iTDP-43^14A^ mice at P5 in the absence of FTLD-like TDP-43 aggregation. (A) Monoclonal antibody to human TDP-43 showed expression at P5 remained restricted to previously characterized regions of hippocampus, cortex and striatum. (B) Western blotting of brain lysate of P5 non-transgenic (NT) and iTDP-43^14A^ demonstrated increased levels of activated caspase 3 in iTDP-43^14A^ mice. (C) Abundant caspase 3 immunoreactivity in the cortex of iTDP-43^14A^ mice that was virtually absent in NT mice, suggestive of elevated cell death in iTDP43^14A^ compared to NT mice. iTDP-43^14A^ mice were also characterized by increased ubiquitin staining in the upper layers of the cortex compared to NT mice, which upon higher magnification appeared to be completely diffuse and cytoplasmic. (D) Immunohistochemistry for hTDP-43 and p403/404 and immunofluorescence using antibodies to total TDP-43 and p409/410 TDP-43. Significant amounts of cytoplasmic hTDP-43 were observed in iTDP-43 mice (arrowheads). Note that this cytoplasmic staining was also observed in NT mice (arrowheads) with antibodies to total TDP-43 (tTDP-43 Ab1) and TDP-43 phosphorylated at 409/410 (p409/410). Scale bars in D = 50 µm.

To examine the potential underlying mechanisms of neurodegeneration in iTDP-43^14A^, we screened for neuropathology consistent with FTLD-TDP at P5, the earliest neurodegenerative time point studied. Immunohistochemical detection of hTDP-43^M337V^ expression using human-specific antibody revealed abundant expression in cortex, hippocampus and striatum (figure 2A). To confirm active cell death, we used antibodies to activated caspase 3. Western blotting of brain lysate revealed low level activated caspase-3 in NT mice, which is compatible with programmed apoptosis during developmental stages [30]; however, this was markedly increased in iTDP-43^14A^ animals (figure 2B). This was confirmed by immunohistochemical detection of extensive activated caspase 3 immunoreactivity in the cortex of iTDP-43^14A^ mice (figure 2C), suggesting extensive cell death at P5 as a result of the expression of M337V TDP-43. We detected no increase in activated caspase-3 by western blot in monogenic tTA mice compared to NT mice, ruling out tTA expression as the cause of this phenotype (figure s2B).

The end-stage neuropathology of FTLD-TDP is characterized by the presence of ubiquitinated, phosphorylated TDP-43 aggregates in the cytoplasm of affected neurons and glia. We used antibodies to ubiquitin, human TDP-43 (hTDP-43), total TDP-43 (tTDP-43 Ab1) and TDP-43 phosphorylated at residues 403/404 (p403/404) and 409/410 (p409/410) to investigate the contribution of aggregation to the cell death observed in P5 iTDP-43^14A^ mice. We qualitatively observed widespread, increased ubiquitin immunoreactivity in the upper layers of the developing cortex (figure 2C). Despite being exclusively cytoplasmic, the staining was diffuse in nature and we found no evidence of ubiquitin positive aggregates characteristic of FTLD-TDP. Furthermore, we found no evidence of TDP-43 aggregation using hTDP-43, tTDP-43, p403/404 or p409/410 antibodies (figure 2D). We did frequently detect cytoplasmic human TDP-43 staining in iTDP-43^14A^ mice within the cortex; however, no aggregates were observed. Both tTDP-43 and p409/410 antibodies showed abundant cytoplasmic reactivity in NT mice, suggesting that this is a spatiotemporally normal distribution (arrowheads in figure 2D). Therefore, cell death at the earliest stage investigated occurred in the complete absence of cytoplasmic or nuclear TDP-43 aggregates of any kind.

### Misprocessing of Human TDP-43 is a Feature of Early Neurodegeneration

The cleavage of TDP-43 into a variety of detergent-insoluble, urea soluble fragments ranging from 17–35 kDa is a hallmark of FTLD-TDP and these fragments are a potential source of neuronal toxicity [1], [5], [6]. Transgenic mice expressing wild type or familial ALS-linked mutant TDP-43 are characterized by the presence of 35 kDa and 25 kDa TDP-43 immunoreactive species [7], [21], [29], [31], [32], [33], [34], [35]. However, the nature of these species is largely unknown. We used a panel of antibodies raised to the N-terminus (residues 3–12), C-terminus (405–414), and central RRM domains of TDP-43 (tTDP-43Ab1 and tTDP-43 Ab2) to determine the extent and nature ofTDP-43 fragmentation in P5 iTDP-43^14A^ mice. To validate the antibodies used, we overexpressed either a C-terminal pathological fragment identified in human cases of FTLD-TDP (TDP-43^208–414^) [36] or the first 280 amino acids of human TDP-43 (TDP-43^1–280^), both with an N-terminal myc tag, in HEK293T cells. Subsequent western analysis of lysate verified that the C-terminal antibody detected TDP-43^208–414^, but not TDP-43^1–280^. Conversely, tTDP-43 Ab2, which is raised to residues 181–198, detected TDP-43^1–280 ^but not TDP-43^208–414^. Human specific antibody and myc antibody detected both. TDP-43 3–12 detected TDP-43^1–280 ^but not the C-terminal fragment (figure s3). These findings are consistent with the antigenic epitopes of these antibodies and epitope mapping studies previously published [37], [38].

**Figure 3:**
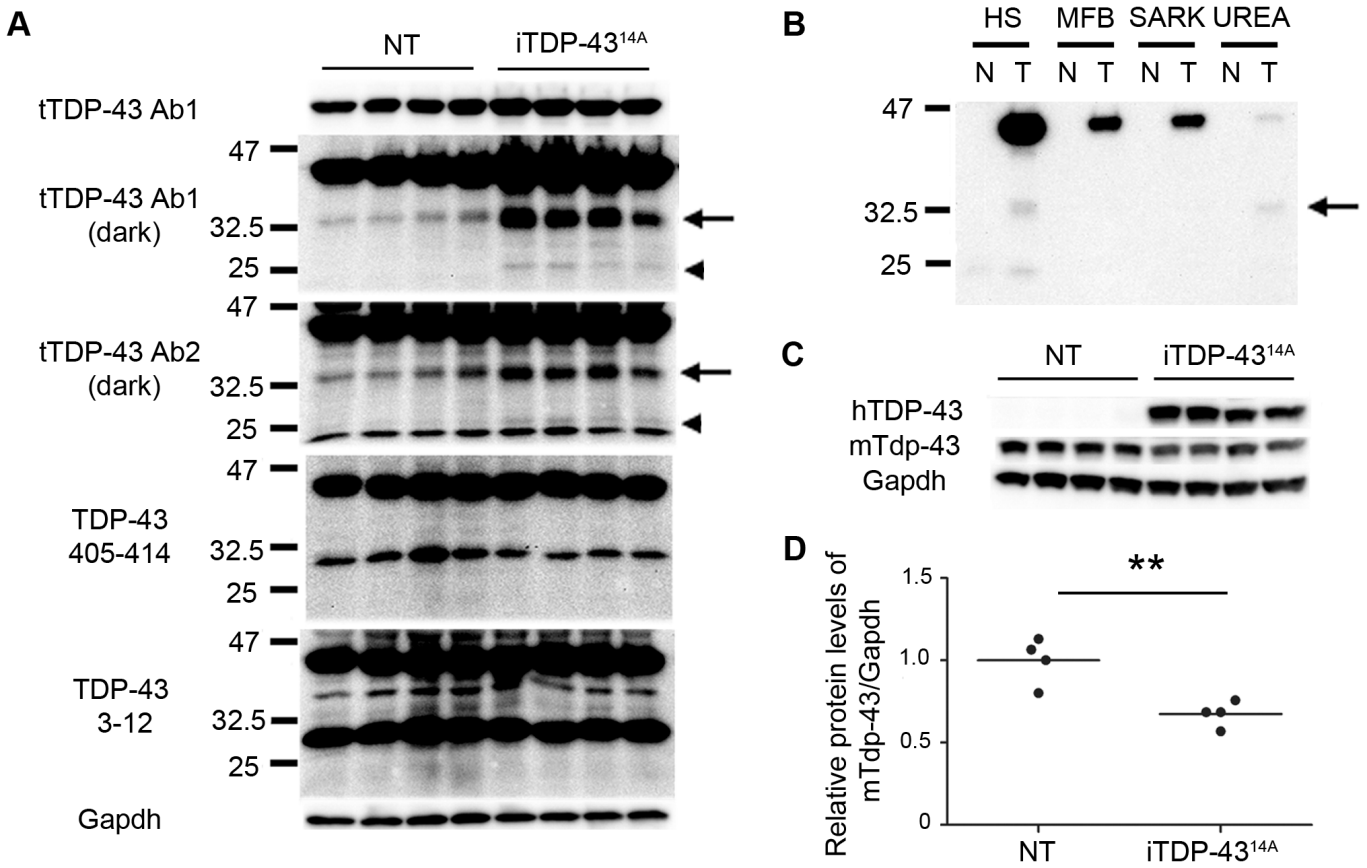
Biochemistry of iTDP-43^14A^ brain lysates at P5. (A) Western blotting using two antibodies to total TDP-43 (tTDP-43 Ab1 and tTDP-43 Ab2) demonstrated increased levels of low molecular weight species at 35 kDa (arrow) and 25 kDa (arrowhead) in iTDP-43^14A^ mice relative to NT mice. These species were not observed using antibodies to the C-terminus (405–414) or N-terminus (3–12) of TDP-43. (B) Western blot analysis of high salt (HS), myelin floatation buffer (MFB), sarkosyl (SARK) and urea fractions using antibody to human TDP-43. Note that human TDP-35 (arrow) is present in the urea fraction but is absent from MFB and SARK fractions, N = non-transgenic, T = iTDP-43^14A^. (C) Antibody to murine Tdp-43 demonstrated reduction of mTdp-43 in brain compared to NT mice. (D) Quantification of blot in (C), **p<0.01, unpaired two tailed *t-*tes*t*.

In SDS soluble fractions of P5 brain lysate from iTDP-43^14A^ mice, we detected species of approximately 35 kDa (TDP-35) and 25 kDa (TDP-25) using total TDP-43 Ab1 and total TDP-43 Ab2 (figure 3A). Both of these antibodies recognize epitopes in the second RNA recognition motif of mTdp-43 and hTDP-43, which is located in the center of TDP-43. However, we failed to detect either of these low molecular weight species using antibodies raised to the C-terminus (residues 405–414) or N-terminus (residues 3–12) of TDP-43 (figure 3A).

To assess the solubility of TDP-43, TDP-35 and TDP-25, we used a series of increasingly stringent buffers to sequentially extract protein from cortical tissue of p5 iTDP-43^14A^ mice. Western blotting of the resulting fractions and analysis using human specific TDP-43 antibody demonstrated that although a proportion of TDP-35 was detergent insoluble but urea soluble, the majority of TDP-35 and TDP-25 are soluble in high salt buffer (figure 3b). Consequently, during early neurodegeneration in iTDP-43^14A^, TDP-43 fragments are derived from a central portion of human TDP-43 and are largely soluble.

Another cardinal feature of FTLD-TDP is loss of nuclear TDP-43, which may contribute to disease via aberrant transcriptional control and/or misprocessing of RNA substrates bound by TDP-43 [2]. In a previously published transgenic line expressing human wild type TDP-43, loss of murine Tdp-43 (mTdp-43) occurred upon human TDP-43 transgene expression and correlated with phenotypic progression, suggesting that loss of mTdp-43 may be analogous to loss of nuclear TDP-43 in human disease [23] despite human transgene expression in the nucleus. Using an antibody specific to mTdp-43, we detected a 33% decrease in the expression of mTdp-43 in iTDP-43^14A^ brain extract compared to NT animals (0.67±0.03, *p* = 0.007 unpaired two tailed *t*-test, figure 3C and D).

### Low Level Misprocessing of TDP-43 is Well Tolerated *in vivo*

Given the lack of an overt neurodegenerative phenotype in the second iTDP-43^8A^ line, we aged iTDP-43^8A^ cohorts to 10 and 25 months. In both cohorts, brain weights of both tTA monogenic and iTDP-43^8A^ mice were reduced 5–7% compared to NT (10 M: NT = 496±6 mg *N* = 5, iTDP-43^8A^ = 462±4 mg *N* = 7, tTA = 464.9±9 mg *N* = 7, *p*<0.05 one way ANOVA Bonferroni *post-hoc* analysis; 25 M: NT = 505±8 mg *N* = 7, iTDP-43^8A^ = 470±7 mg, tTA = 473±7 mg, *p*<0.05 one way ANOVA Bonferroni *post-hoc* analysis, figure s4) and there was decreased volume of the dentate gyri, a previously documented observation that likely reflects a phenotype driven by this tTA line rather than the expression of the TDP-43 transgene [32]. With this exception, we observed no phenotype in these animals as assessed by gross weight or brain morphology using hematoxylin and eosin staining (figure s4). We verified that expression of tTA did not alter the levels of endogenous mTdp-43 or the abundance of lower molecular weight TDP species (figure s4). Therefore, although tTA expression results in reduced brain weight in older mice, it does not alter TDP-43 biochemistry.

**Figure 4:**
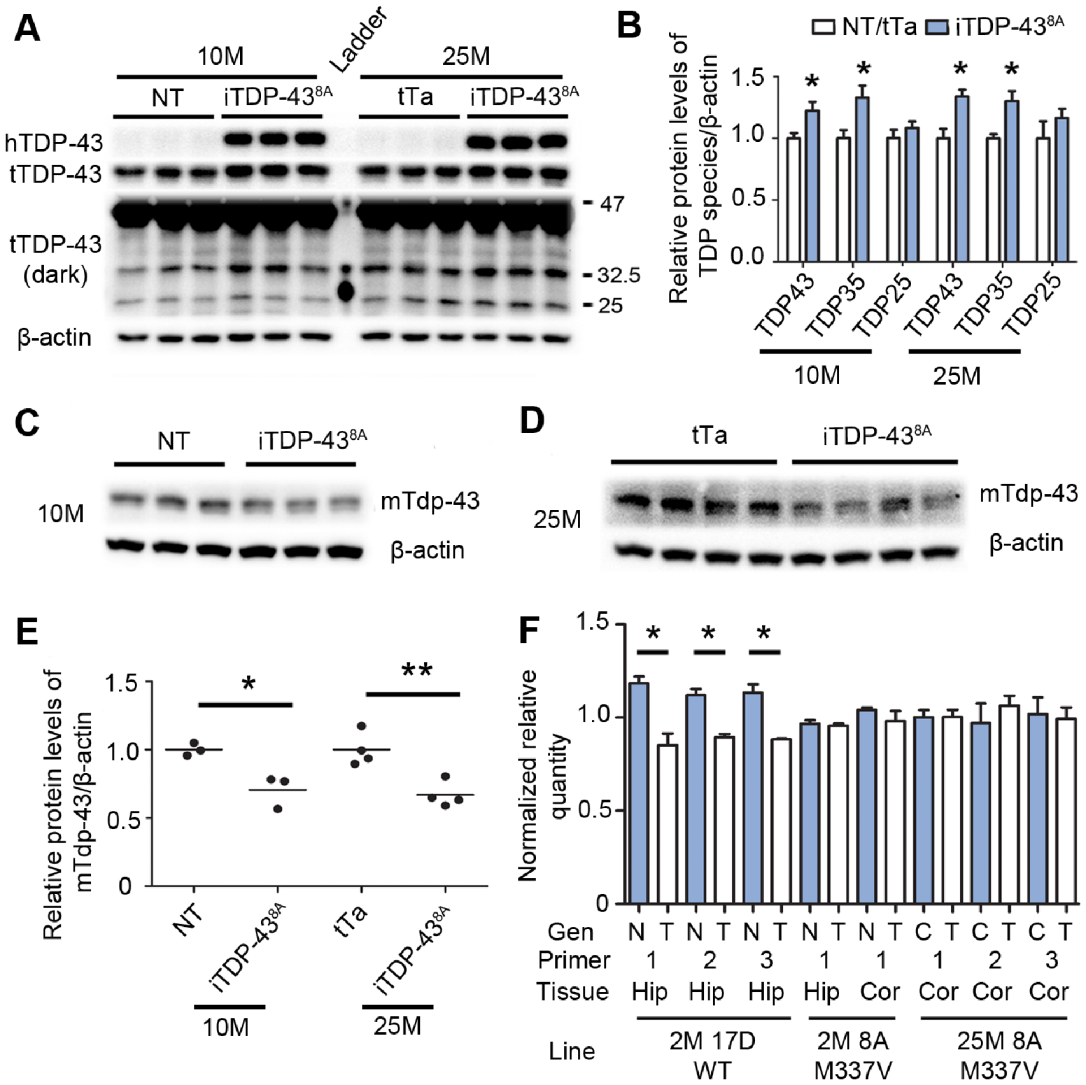
Loss of murine Tdp-43 and increased TDP-35 in 25 month (25 M) iTDP-43^8A^. (A) Analysis of transgene expression in 10 M and 25 M cohorts using hTDP-43 and tTDP-43 Ab2 antibodies, β-actin for loading. (B) Densitometric quantitation of TDP-43, TDP-35 and TDP-25 from blot in (A), represented as relative levels of TDP species following sample normalization to β-actin loading controls. (C) mTdp-43 expression in 10 M iTDP-43^8A^, β-actin loading control. (D) mTdp-43 expression in 25 M iTDP-43^8A^, β-actin loading control. (E) Densitometric quantitation of reduction in mTdp-43 protein levels from blots in (C) and (D), represented as relative levels of mTdp-43 following sample normalization to β-actin loading controls. (F) Quantitative PCR analysis of *Tardbp* transcript in 2 month old transgenic (T) iTDP-43^17D^ mice hippocampus (HIP) using *Tardbp* primers 1, 2 and 3 detected significant reduction in murine Tdp-43 mRNA levels compared to nontransgenic (N) mice and acted as a positive control for our methodology (*N* = 3 per genotype). No reduction in *Tardbp* mRNA was detected in the cortex (COR) and hippocampus of 2 month (2 M) iTDP-43^8A^ mice relative to nontransgenic mice using murine-specific *Tardbp* primer pair 1 (*N* = 3 per genotype). No downregulation of *Tardbp* was observed in the cortex of 25 M iTDP-43^8A^ (*N* = 3) relative to control (C) mice (*N* = 4), using *Tardbp* primers 1, 2 or 3. Values shown as Normalized Relative Quantity, see Materials and Methods for further details. Control at 25 M consisted of 2 non-transgenic and 2 tTA mice. In graphs (B),(E) and (F): *p<0.05, **p<0.01, unpaired *t-*test, error bars are SEM.

Due to the lack of an overt phenotype, we hypothesized that any TDP-43 biochemical alterations in the iTDP-43^8A^ line may be small in magnitude. In order to increase detection of low level alterations over background, we used lysate of dissected cortex as opposed to brain lysate, thus enriching hTDP-43^M337V^ expressing regions and reducing noise from regions not expressing the transgene. Expression levels of the hTDP-43^M337V^ transgene were exceptionally low in both the 10 and 25 month iTDP-43^8A^ cohorts. Western blotting and densitometry analysis of cortical extracts using tTDP-43 Ab2 demonstrated ∼1.25 fold overexpression of TDP-43 compared to NT mice (10 M = 1.22±0.07, 25 M = 1.33±0.05, figure 4A, quantification in 4B). In both cohorts, increased levels of TDP-35 but not TDP-25 was observed relative to control mice (10 M TDP-35 = 1.32±0.09, *p* = 0.049; 10 M TDP-25 = 1.08±0.05, *p* = 0.396; 25 M TDP-35 = 1.30±0.08, *p* = 0.026; 25 M TDP-25 = 1.16±0.16 *p* = 0.353, figure 4A and B). We also measured levels of mTdp-43 protein in cortical extracts of both cohorts and found it to be downregulated in iTDP-43^8A^ compared to controls (10 M = 0.71±0.07, *p* = 0.017; 25 M = 0.67±0.05, *p* = 0.005; figure 4C, D and E). Given that TDP-43 autoregulates the abundance of *Tardbp* mRNA *in vivo* and *in vitro* [2], [39], we used quantitative PCR to examine levels of murine *Tardbp* transcript. Using three different primer pairs to *Tardbp*, we found no evidence in the cortex of 25 month old animals for the downregulation of murine *Tardbp* transcript in iTDP-43^8A^ mice compared to control animals (figure 4F). Additionally, we failed to detect downregulation in the cortex and hippocampus of 2 month old iTDP-43^8A^ animals (figure 4F). Seeking to validate our methodology, we analyzed hippocampal RNA from a line of transgenic mice overexpressing human wild type TDP-43, iTDP-43^17D^, that have previously been reported to express reduced *Tardbp* mRNA [24]. We observed a statistically significant reduction in *Tardbp* using all three primer pairs (figure 4F). These data suggest that additional mechanisms may exist *in vivo* that regulate Tdp-43 levels independent of *Tardbp* total transcript levels.

**Figure 5:**
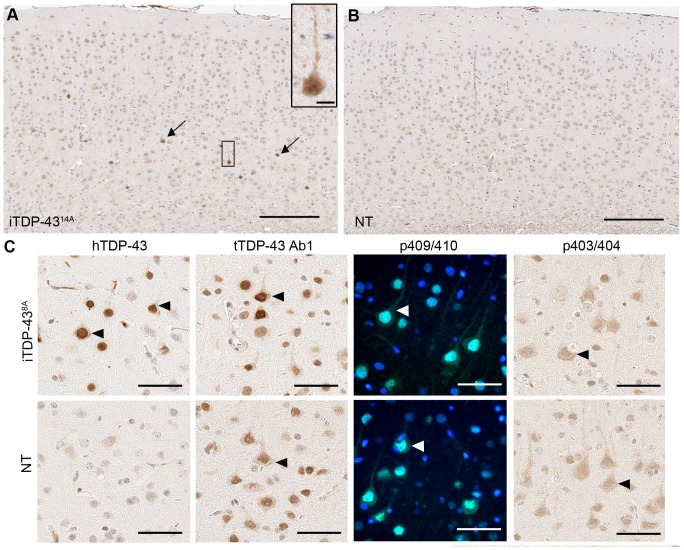
Neuropathology of 25 month old iTDP-43^8A^ mice. Immunohistochemical detection of ubiquitin revealed rare cells bearing increased ubiquitin staining in the cortex of iTDP-43^8A^ mice (arrows, A) that was absent in NT animals (B, scale bar = 200 µm). Staining was detected in both nucleus and cytoplasm of affected cells (inset in A, scale bar = 10 µm). (C) In iTDP-43^8A^ animals hTDP-43 was predominantly nuclear, some cells displaying cytoplasmic localization without aggregation. Cytoplasmic localization was observed in NT and iTDP-43^8A^ mice using antibodies to total TDP-43 (tTDP-43 Ab1) and phosphorylated forms of TDP-43 (p403/404, p409/410).

To determine the neuropathogical consequences of low level M337V TDP-43 expression in the older 25 M cohort, we again examined markers of FTLD-TDP pathology. The cortex of 25 M old iTDP-43^8A^ animals was characterized by rare cells bearing intense ubiquitin reactivity, predominantly in the lower cortical layers (figure 5A and B). This increased staining for ubiquitin was both nuclear and cytoplasmic (inset, figure 5A), which contrasts with the exclusively cytoplasmic distribution in P5 iTDP-43^14A^ mice. As in P5 iTDP-43^14A^ mice, we found no evidence of TDP-43 aggregation using hTDP-43, tTDP-43, p403/404 or p409/410 antibodies (figure 5C). Although we observed neurons in iTDP-43 mice bearing hTDP-43 reactivity in the cytoplasm, numerous cells were observed in both NT and iTDP-43 mice with cytoplasmic tTDP-43, p409/410 and p403/404 (arrowheads in figure 5C). In conjunction with our biochemical analysis, this evidence suggests that an approximately 30% reduction in mTdp-43 in the presence of low level expression of hTDP-43 and simultaneous low level increases in TDP-35 result in relatively minor neuropathological abnormalities and are well tolerated even over long time spans.

## Discussion

TDP-43 plays a major role in the pathogenesis of FTLD-TDP. Mutations in *TARDBP* cause some cases of TDP-43-proteinopathy in amyotrophic lateral sclerosis. Numerous transgenic rodents overexpressing wild type or mutant TDP-43 have been reported that have developed profound neurodegenerative phenotypes, early lethality and gait abnormalities. Some features of FTLD-TDP and/or ALS have been recapitulated in these transgenic lines including aggregated, hyperphosphorylated TDP-43, and misprocessing of TDP-43 into lower molecular weight species.

In this paper, we generated two lines of mice expressing human TDP-43 containing the familial M337V mutation driven by the CaMKIIα promoter to determine the effects of mutant TDP-43 expression in the forebrain. Of note, neither iTDP-43 line exhibited overt phenotype comparable to previous reports (early lethality, weight loss, limb weakness or gait disturbances). As transgenic lines driven by the CaMKIIα promoter or bearing the M337V mutation have already been reported that do exhibit these phenotypes [21], [24], [33], the absence here of early lethality or gait disturbance cannot be entirely explained by these variables alone, suggesting that a combination of these and other factors such as timing of transgene expression and background strain govern ALS-like phenotypes.

The human neuropathology of FTLD-TDP - consisting of loss of nuclear TDP-43, TDP-43 aggregation, phosphorylation and fragmentation to C-terminal lower molecular weight species - has driven hypotheses as to TDP-43 toxicity both in human disease and in mice expressing transgenic TDP-43. Herein, we found that early neurodegeneration in the iTDP-43^14A^ mouse line occurred in the complete absence of aggregation. This observation is consistent with previous research demonstrating that aggregation does not appear to be a major driver of cell death in TDP-43 transgenic animals [22], [23]. However, we cannot rule out that aggregates may be neurotoxic, particularly in lines in which they feature more prominently [21], [24]. In contrast, downregulation of endogenous mTdp-43 is a feature of early degeneration in iTDP-43^14A^, and this has been implicated elsewhere as a potential driver of neurodegeneration where it correlates with phenotypic progression [23]. However, data from our second line iTDP-43^8A^ – in which we observed downregulation of mTdp-43 of 30% until 25 months of age, the oldest age examined – indicates that if loss of murine Tdp-43 is capable of driving aggressive neurodegenerative phenotypes, a critical threshold must be reached. Interestingly, we failed to detect downregulation of murine *Tardbp* transcript in either young or aged iTDP-43^8A^ cortex despite the reduction in mTdp-43 protein. This contrasts with findings from our lab and elsewhere demonstrating *in vivo,* hTDP-43 autoregulated reduction in *Tardbp* as the underlying mechanism of reduced Tdp-43 [2], [20], [33]. This finding may be indicative of other post-transcriptional autoregulatory activity (for example, TDP-43 mediated translational repression [40]) or that the subtle phenotype observed in aged iTDP-43^8A^ mice is sufficient to cause a measurable reduction in Tdp-43.

A potential additional source of neurotoxicity in iTDP-43^14A^ may be fragmentation of TDP-43 to the low molecular weight species TDP-35 and TDP-25. At the earliest phenotypic time point studied here, we observed accumulation of these species at least partially derived from the human TDP-43 transgene and found evidence of detergent insoluble, urea soluble TDP-35. Although these species may be inherently neurotoxic and contribute to cell death in iTDP-43^14A^, we also determined that they are derived from a central portion of TDP-43 and are thus may not be biochemically identical to the C-terminally derived fragments of human disease. Consequently, we cannot be certain that any neurotoxicity these species invoke is relevant to human pathology. More study is required to determine if these fragments are identical to those almost universally present in other TDP-43 transgenic animals. Elsewhere, these fragments have been described as C-terminal in nature, based on the use of antibodies raised to the C-terminal 154 residues of TDP-43 [20]. To our knowledge the epitope of this polyclonal antibody has not been mapped. However, as a large proportion of the immunogen corresponds to the center of TDP-43, it is possible this ‘C-terminal’ antibody detects centrally derived fragments also.

Consequently, our data here add to prior studies suggesting that neurodegenerative phenotypes in existing TDP-43 transgenic animals might be influenced by a number of factors depending on the transgenic line, and that it may prove difficult to separate toxicity of potentially disease relevant phenomena from one another or from global RNA dysregulation that occurs upon mutant TDP-43 overexpression [41]. Additionally, the phenotype in iTDP-43^14A^ is aggressive and occurs prior to full development of the brain, an effect that we have documented in a previous line constitutively expressing wild type TDP-43 and that may be the case in several other high expressing transgenic lines with early phenotypes [20], [22], [24], [29], [33]. This clearly makes for a poor model of an age-related neurodegenerative condition. While existing TDP-43 transgenic mice will undoubtedly provide insight into TDP-43 function and in some instances can be used as investigative tools, the field still lacks *in vivo* models that faithfully recapitulate age dependent accumulation of disease relevant TDP-43 pathology.

## Supporting Information

Figure S1 TDP-43 antibodies used in this study. N-terminal, C-terminal, mTDP-43 and tTDP-43 Ab2 are raised to the indicated epitopes. hTDP-43 and tTDP-43 Ab1 antibodies have been mapped to the indicated epitopes in previous studies [37], [38]. Murine Tdp-43 specific antibody was raised as previously described [42].
(TIF)

Figure S2 tTA expression is not responsible for iTDP-43^14A^ phenotypes. (A) Brain weights of NT, monogenic hTDP-43^14A^ and monogenic tTA mice at P5 and 2 months of age were identical. (B) Western analysis of P5 brain lysate demonstrated no increase in activated caspase 3 in tTA only mice compared to NT mice. However, elevated activated caspase 3 was observed in iTDP-43^14A^.
(TIF)

Figure S3 Validation of TDP-43 antibody specificity. Western analysis of lysate from HEK-293T cells transfected with empty vector, myc-hTDP-43^1–280^ or myc-hTDP-43^208–414^. Blots were probed with the indicated antibodies.
(TIF)

Figure S4 Expression of tTA trangene does not affect TDP-43 metabolism. (A) Brain weights of iTDP-43^8A^ mice covering the postnatal period and aged cohorts. No significant difference in weight was observed in the time period to 2 months; tTA and iTDP-43 mice in 10 month and 25 month cohorts showed a small, significant decrease relative to NT mice (*N*(P0) = 4 per genotype; *N*(P12) = 3 per genotype; *N*(P21) = 4 per genotype; *N*(2 M) = 5 per genotype; *N*(10 M) = 5 NT, 8 iTDP-43, 7 tTA; *N*(25 M) = 7 NT, 7 iTDP-43, 5 tTA. Error bars are SD, *p<0.05, one way ANOVA, Bonferroni *post hoc*). (B) Hematoxylin and eosin staining of 25 M cohorts from line iTDP-43^8A^ show no gross morphological differences between tTA and iTDP-43^8A^ cortex. There was a trend toward smaller dentate gyri in tTA mice compared to NT mice. (C) Long exposure of western blot of 11 M NT and tTA cortical lysates using antibody to total TDP-43 (tTDP-43 Ab2), mouse specific TDP-43 antibody was used to probe same lysates, β-actin for loading. (D) Densitometric quantitation of blots in (C) confirmed no change in TDP-43, TDP-35 or TDP-25 in tTA mice versus NT mice; values are represented as fold change relative to NT mice following sample normalization to β-actin loading controls.
(TIF)

Table S1 Primary antibodies used in this study.
(DOCX)
