# Supplementary figures and images for: Divergent Phenotypes in Mutant TDP-43 Transgenic Mice Highlight Potential Confounds in TDP-43 Transgenic Modeling
Source: PLoS One. 2014 Jan 22;9(1):e86513. doi: 10.1371/journal.pone.0086513 (PMC3899264; doi:10.1371/journal.pone.0086513)

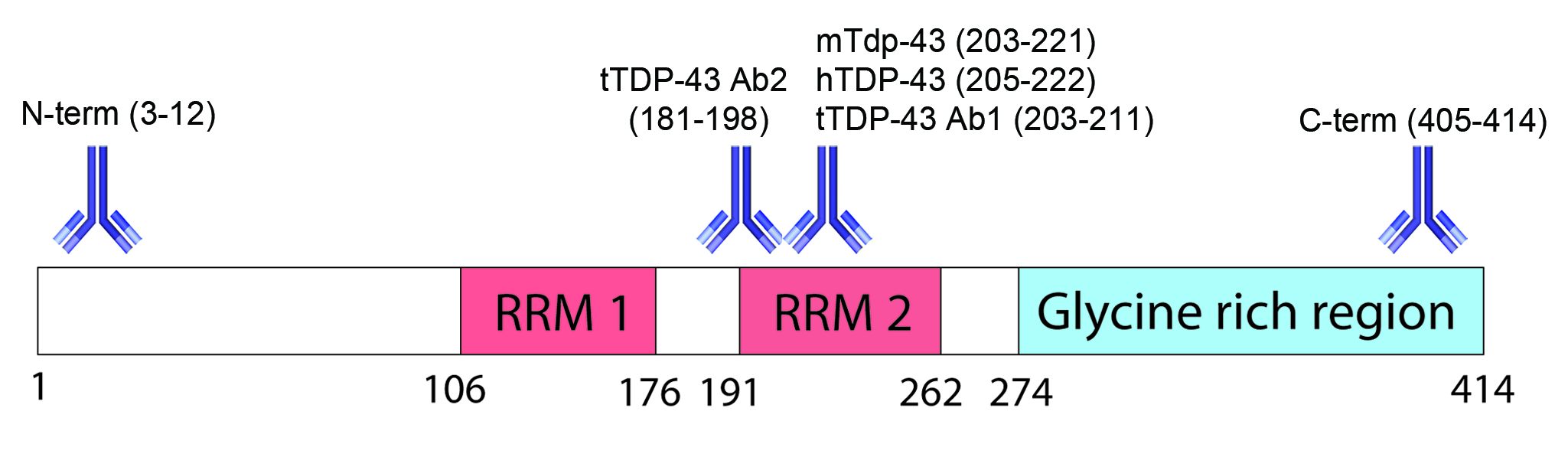

Supplement: Figure S1 — TDP-43 antibodies used in this study. N-terminal, C-terminal, mTDP-43 and tTDP-43 Ab2 are raised to the indicated epitopes. hTDP-43 and tTDP-43 Ab1 antibodies have been mapped to the indicated epitopes in previous studies [37], [38]. Murine Tdp-43 specific antibody was raised as previously described [42]. (TIF) [file pone.0086513.s001.tif]

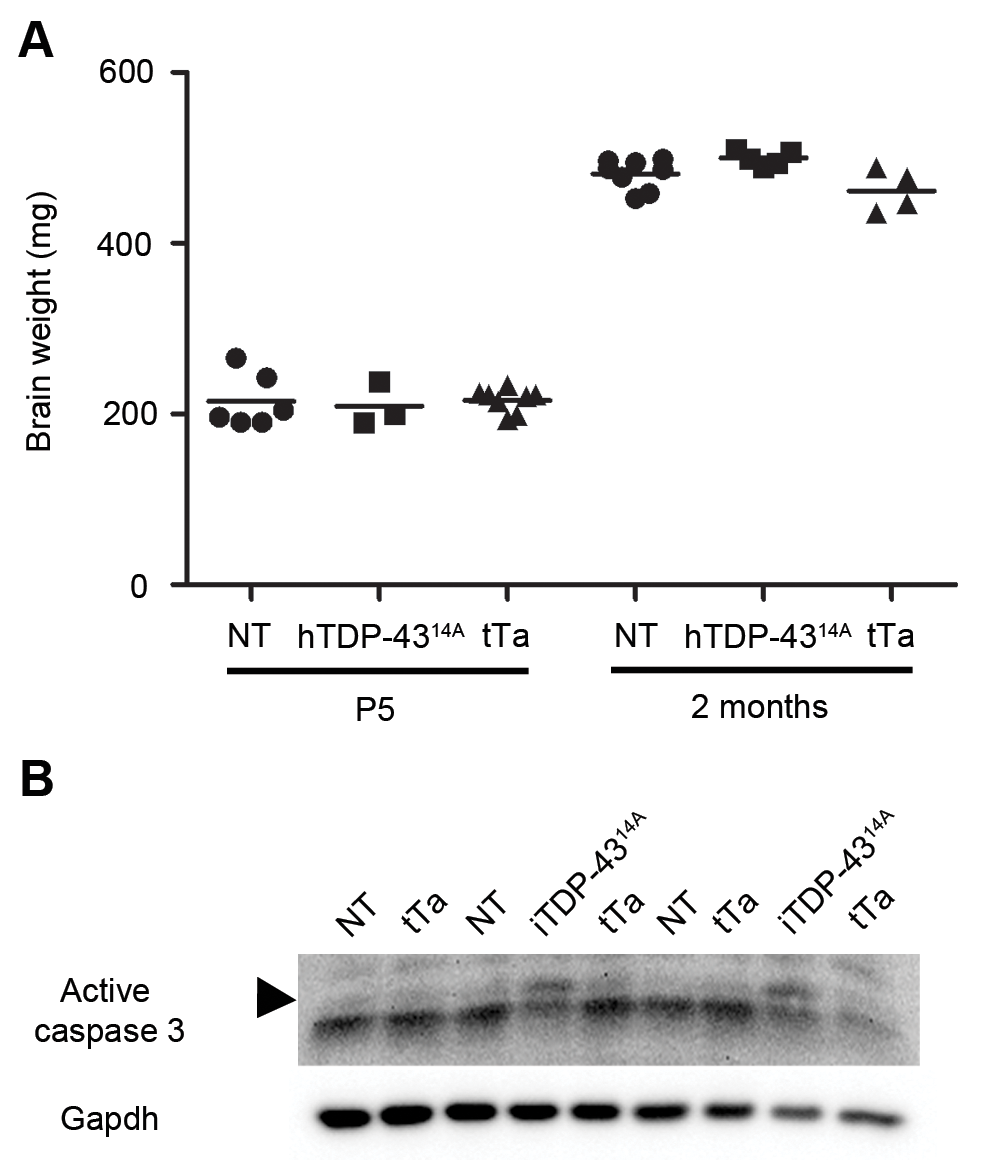

Supplement: Figure S2 — tTA expression is not responsible for iTDP-4314A phenotypes. (A) Brain weights of NT, monogenic hTDP-4314A and monogenic tTA mice at P5 and 2 months of age were identical. (B) Western analysis of P5 brain lysate demonstrated no increase in activated caspase 3 in tTA only mice compared to NT mice. However, elevated activated caspase 3 was observed in iTDP-4314A. (TIF) [file pone.0086513.s002.tif]

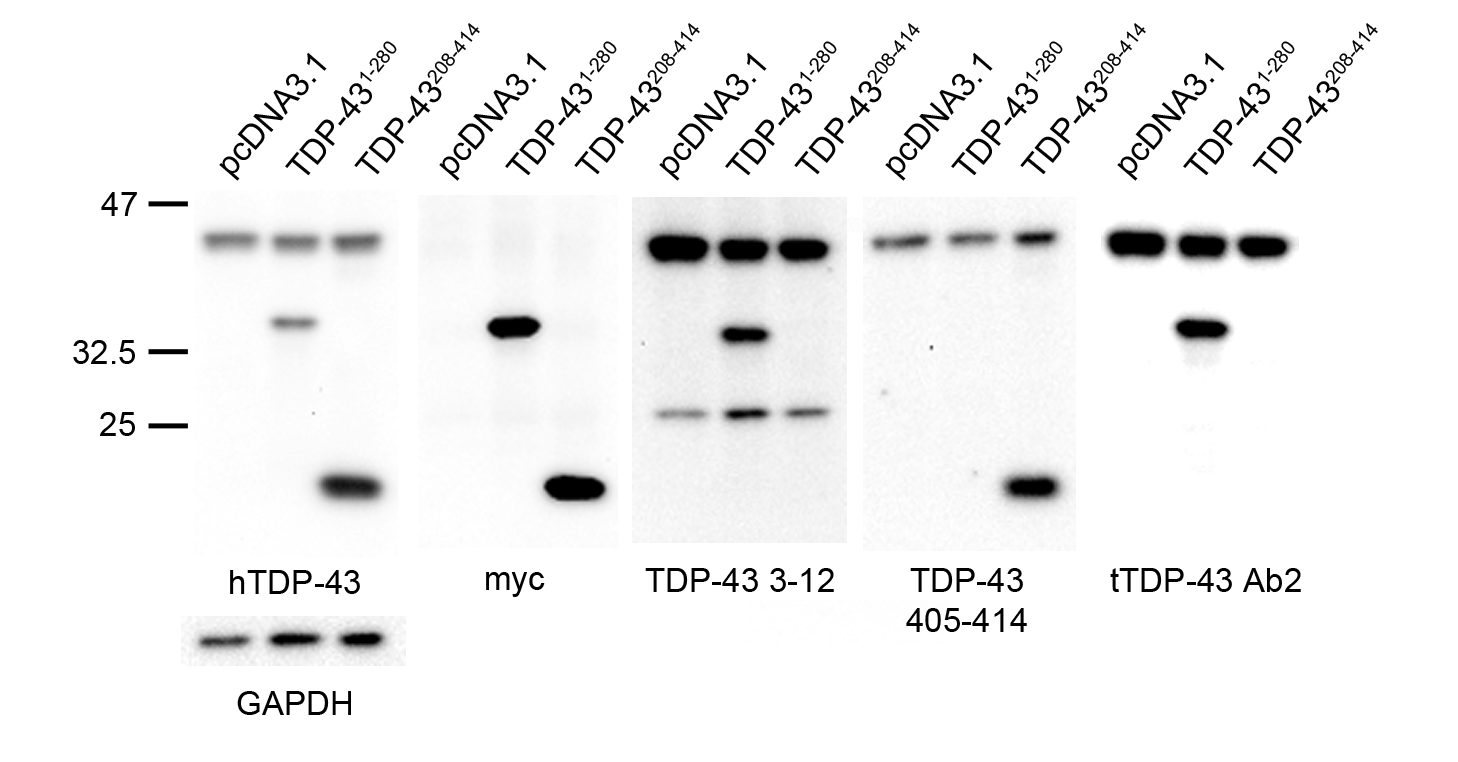

Supplement: Figure S3 — Validation of TDP-43 antibody specificity. Western analysis of lysate from HEK-293T cells transfected with empty vector, myc-hTDP-431–280 or myc-hTDP-43208–414. Blots were probed with the indicated antibodies. (TIF) [file pone.0086513.s003.tif]

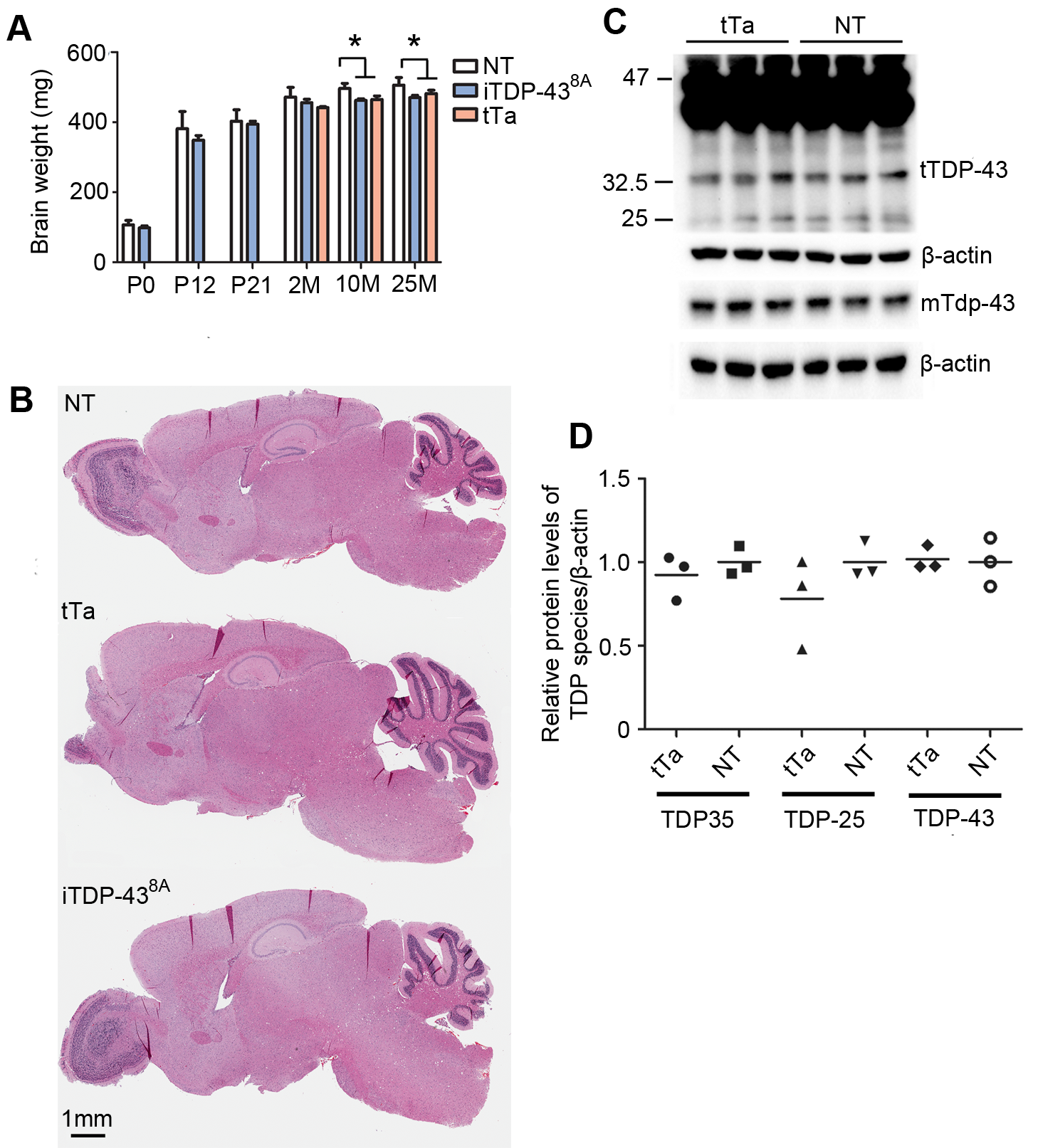

Supplement: Figure S4 — Expression of tTA trangene does not affect TDP-43 metabolism. (A) Brain weights of iTDP-438A mice covering the postnatal period and aged cohorts. No significant difference in weight was observed in the time period to 2 months; tTA and iTDP-43 mice in 10 month and 25 month cohorts showed a small, significant decrease relative to NT mice (N(P0) = 4 per genotype; N(P12) = 3 per genotype; N(P21) = 4 per genotype; N(2 M) = 5 per genotype; N(10 M) = 5 NT, 8 iTDP-43, 7 tTA; N(25 M) = 7 NT, 7 iTDP-43, 5 tTA. Error bars are SD, *p<0.05, one way ANOVA, Bonferroni post hoc). (B) Hematoxylin and eosin staining of 25 M cohorts from line iTDP-438A show no gross morphological differences between tTA and iTDP-438A cortex. There was a trend toward smaller dentate gyri in tTA mice compared to NT mice. (C) Long exposure of western blot of 11 M NT and tTA cortical lysates using antibody to total TDP-43 (tTDP-43 Ab2), mouse specific TDP-43 antibody was used to probe same lysates, β-actin for loading. (D) Densitometric quantitation of blots in (C) confirmed no change in TDP-43, TDP-35 or TDP-25 in tTA mice versus NT mice; values are represented as fold change relative to NT mice following sample normalization to β-actin loading controls. (TIF) [file pone.0086513.s004.tif]
