# Supplementary material for: Divergent Phenotypes in Mutant TDP-43 Transgenic Mice Highlight Potential Confounds in TDP-43 Transgenic Modeling
Source: PLoS One. 2014 Jan 22;9(1):e86513. doi: 10.1371/journal.pone.0086513 (PMC3899264; doi:10.1371/journal.pone.0086513)
Supplement: Table S1 — Primary antibodies used in this study. (DOCX) [file pone.0086513.s005.docx]

| **Supplementary Table 1** Primary Antibodies | | |  |  |  |  |
| --- | --- | --- | --- | --- | --- | --- |
|  |  |  |  |  |  |  |
| **Antibody** | **Description** | **Catalogue #** | **Vendor** | **WB** | **IHC** | **IF** |
| mouse monoclonal | human TDP-43 | 2E2-E3 | Novus | 1:2000 | 1:1500 |  |
| TDP-43 | specific |  | Biologicals |  |  |  |
|  |  |  |  |  |  |  |
| rabbit polyclonal | mouse TDP-43 | In house | In house | 1:1000 |  |  |
| TDP-43 | specific |  |  |  |  |  |
|  |  |  |  |  |  |  |
| rabbit polyclonal | total TDP-43 | 10782-2-AP | Proteintech | 1:2000 | 1:1500 | 1:1000 |
| TDP-43 | Ab 1 |  |  |  |  |  |
|  |  |  |  |  |  |  |
| rabbit polyclonal | total TDP-43 | T1705 | Sigma | 1:2000 |  |  |
| TDP-43 | Ab 2 |  |  |  |  |  |
|  |  |  |  |  |  |  |
| rabbit phospho- | phosphoserine | TIP-PTD-P05 | Cosmo Bio |  | 1:1000 |  |
| TDP-43 (pS403-404) | 403/404 |  |  |  |  |  |
|  |  |  |  |  |  |  |
| rabbit phospho- | phosphoserine | TIP-PTD-P02 | Cosmo Bio |  |  | 1:1000 |
| TDP-43 (pS409/410) | 409/410 |  |  |  |  |  |
|  |  |  |  |  |  |  |
| rabbit polyclonal | N-terminus | TIP-TD-P07 | Cosmo Bio | 1:1000 |  |  |
| TDP-43 3-12 | TDP-43 |  |  |  |  |  |
|  |  |  |  |  |  |  |
| rabbit polyclonal | C-terminus | TIP-TD-P09 | Cosmo Bio | 1:1000 |  |  |
| TDP-43 405-414 | TDP-43 |  |  |  |  |  |
|  |  |  |  |  |  |  |
| mouse monoclonal |  | 05-944 | Chemicon |  | 1:60000 |  |
| ubiquitin |  |  |  |  |  |  |
|  |  |  |  |  |  |  |
| cleaved caspase 3 |  | 9664 | Cell | 1:1000 | 1:100 |  |
|  |  |  | Signalling |  |  |  |
|  |  |  |  |  |  |  |
| Gapdh |  | A8634OH | Biodesign | 1:5000 |  |  |
|  |  |  |  |  |  |  |
| β-actin |  | A1978 | Sigma | 1:5000 |  |  |
|  |  |  |  |  |  |  |
| myc |  | M4439 | Sigma | 1:5000 |  |  |
